# Diel Dynamics of Field Responses to UV and Photosynthetic Radiation in the Aquatic Liverwort *Jungermannia eucordifolia* and Their Potential Use in UV Biomonitoring

**DOI:** 10.3390/plants15162446

**Published:** 2026-08-12

**Authors:** Alberto-José Parada-Siles, Rafael Tomás-Las-Heras, Laura Monforte, Encarnación Núñez-Olivera, Javier Martínez-Abaigar

**Affiliations:** Facultad de Ciencia y Tecnología, Universidad de La Rioja, 26006 Logroño, Spain; alparada@unirioja.es (A.-J.P.-S.); rafael.tomas@unirioja.es (R.T.-L.-H.); laura.monforte@unirioja.es (L.M.); javier.martinez@unirioja.es (J.M.-A.)

**Keywords:** bryophytes, liverworts, mountain streams, diel changes, field conditions, hydroxycinnamic acids, UV-absorbing compounds, ultraviolet radiation

## Abstract

We investigated diel physiological responses of the leafy aquatic liverwort *Jungermannia eucordifolia* under natural field conditions during two consecutive days. Photosynthetically active radiation (PAR), UV-A and UV-B radiation, together with water temperature, were monitored throughout the study. Physiological responses included: (1) chlorophyll fluorescence assessed through steady-state fluorescence parameters and fast chlorophyll fluorescence induction kinetics (OJIP approach); (2) UV-absorbing phenolic compounds in the methanol-soluble and methanol-insoluble fractions, mainly representing vacuolar and cell wall-bound compounds, respectively, thereby covering different functional modalities of UV protection; both the bulk UV absorption capacity of UV-absorbing compounds and seven individual phenolic compounds were analyzed; and (3) DNA damage. Photosynthetic performance parameters, including the effective photochemical quantum yield of photosystem II (Φ_PSII_), the maximum photochemical quantum yield of photosystem II (F_v_/F_m_), and the Performance Index (PI), together with the photoprotection parameter non-photochemical quenching (NPQ), showed pronounced diel fluctuations closely associated with changes in ambient irradiance. Φ_PSII_, F_v_/F_m_, and PI were negatively related to irradiance, whereas NPQ exhibited the opposite trend. These responses were consistent with those previously observed in this liverwort under controlled laboratory conditions, suggesting dynamic photoinhibition accompanied by efficient photoprotection of photosystem II (PSII) against excess radiation, probably involving the xanthophyll cycle. Nevertheless, the strong covariation among PAR, UV-A, and UV-B wavebands prevented a clear distinction of their individual contributions. In contrast to chlorophyll fluorescence parameters, most variables associated with UV photoprotection through UV-absorbing compounds did not display consistent diel patterns under field conditions, although some individual compounds showed significant temporal fluctuations. This finding contrasted with previous laboratory experiments, where several UV-absorbing compounds increased under enhanced UV-B. DNA damage was not detected in any sample, consistent with previous field studies conducted under ambient UV-B levels but contrasting with experiments using enhanced UV-B exposure under either field or laboratory conditions. These results contribute to a better understanding of short-term physiological dynamics in aquatic bryophytes and may help improve UV biomonitoring protocols based on these organisms.

## 1. Introduction

Bryophytes are important components of many terrestrial and aquatic ecosystems, where they are exposed to fluctuating levels of photosynthetic (photosynthetically active radiation, PAR), ultraviolet (UV), and infrared wavebands, that elicit a wide range of physiological responses. In particular, bryophyte responses to PAR and UV radiation are of evolutionary interest to understand the process of plant terrestrialization, since increases in both factors represented novel environmental pressures in the water-to-land-transition [1]. In general, the effects of UV radiation are more damaging than those of PAR, and consequently a number of field studies have evaluated bryophyte responses to UV over different temporal scales, including interannual [2], seasonal [2,3,4], day-to-day [5,6,7], and diel [3,8,9,10,11,12] scales. Under field conditions, UV responses are influenced by other environmental factors, such as temperature, water availability, and PAR [2]. These interactions may complicate the interpretation of the results obtained. Nevertheless, field studies are ecologically more relevant than laboratory studies because they provide a stronger basis to better establish the real behaviour of plants in response to environmental factors. In particular, field studies on diel physiological dynamics provide valuable information on the short-term responsiveness to naturally fluctuating environmental conditions that cannot be fully reproduced in the laboratory.

Despite their ecophysiological importance, there is a deficit of field studies addressing diel physiological and UV-related changes in aquatic bryophytes. In fact, the only study of this type involving an aquatic bryophyte (*Riella helicophylla*) was conducted under controlled conditions [9]. Moreover, the scarcity of field experiments is particularly evident in bryophytes from flowing waters such as mountain streams, where the present study was conducted. Experiments in mountain streams are logistically more complex than those performed in stagnant waters (the typical habitat of *Riella helicophylla*) or terrestrial environments (where the remaining studies on diel changes were carried out), because of the inherent dynamic nature of these ecosystems. Indeed, such habitats are characterized by relatively rapid changes in water velocity, discharge, oxygen concentration, carbon sources, and mineral nutrients, as well as by streambed heterogeneity, turbulence, and the transport of materials that may cause physical damage to the phytobenthos. Further, compared with terrestrial environments, physiological measurements are more challenging to perform underwater. Consequently, it is not surprising that studies in flowing waters are much less common than those conducted in more stable environments.

It is also noteworthy that, among bryophytes, only one study investigating diel changes in the responses to UV radiation has been conducted on liverworts [9], whereas all the remaining studies focused on mosses [3,8,10,11,12]. Furthermore, the sole study devoted to liverworts examined a thalloid species [9], meaning that diel UV-related responses have never been investigated in leafy liverworts.

Regarding the physiological variables assessed in the studies on diel changes mentioned above, steady-state chlorophyll fluorescence was one of the most commonly used approaches [8,9,10,11,12], as this powerful technique has proven extremely useful for evaluating the effects of a wide range of environmental factors and the possible associated physiological stress [13]. However, fast chlorophyll fluorescence transients (OJIP) allow the calculation of derived parameters such as the Performance Index (PI), which has been shown to be highly sensitive to adverse conditions including excess light, harmful temperatures, drought, etc. [14,15]. Therefore, both chlorophyll fluorescence techniques were applied in the present study. To our knowledge, this is the first report applying the OJIP approach to a leafy liverwort under field conditions.

Other variables have also been used to evaluate diel UV responses in bryophytes, including photosynthetic pigments, DNA damage, and UV-absorbing compounds. Regarding UV-absorbing compounds, previous studies relied exclusively on measurements of the bulk UV absorbance of plant extracts, which integrates the absorbance of all phenolic compounds present in the extract [3,9,11,12]. Consequently, these studies neither distinguished between methanol-soluble and methanol-insoluble fractions nor considered the responses of individual compounds. Differentiating between the methanol-soluble (mainly vacuolar) and methanol-insoluble (mainly cell wall-bound) fractions is important because each may represent a different modality of protection. In particular, cell wall-bound compounds may provide a more spatially uniform UV screen than intracellular compounds [16]. Furthermore, the analysis of individual UV-absorbing compounds complements the overall estimation of UV protection based on bulk UV absorbance, since each compound may respond differently to UV radiation [17]. Overall, UV-absorbing compounds can function both as UV-screens and as antioxidants [18].

The aim of this study was to assess diel changes in the physiological and UV-protective responses of the leafy aquatic liverwort *Jungermannia eucordifolia* Schljakov. The study was conducted over two consecutive days under field conditions. Environmental variables, including PAR, UV-A and UV-B radiation, together with water temperature, were monitored throughout the study. The response variables analyzed were chlorophyll fluorescence (using both steady-state parameters and fast induction kinetics), UV-absorbing compounds (differentiating between methanol-soluble and methanol-insoluble fractions and, in each case, analyzing both the bulk UV absorption capacity of UV-absorbing compounds and several individual compounds), and DNA damage. Given the importance of aquatic bryophytes in UV biomonitoring [1,2], a better understanding of diel changes in their UV responses could contribute to the development of improved monitoring protocols. Therefore, particular emphasis was placed on evaluating the temporal behaviour of potential UV biomarkers, such as *p*-coumaroylmalic acid and other phenolic compounds [1,2].

## 2. Results

### 2.1. Environmental Conditions During the Sampling Period

**Figure 1:**
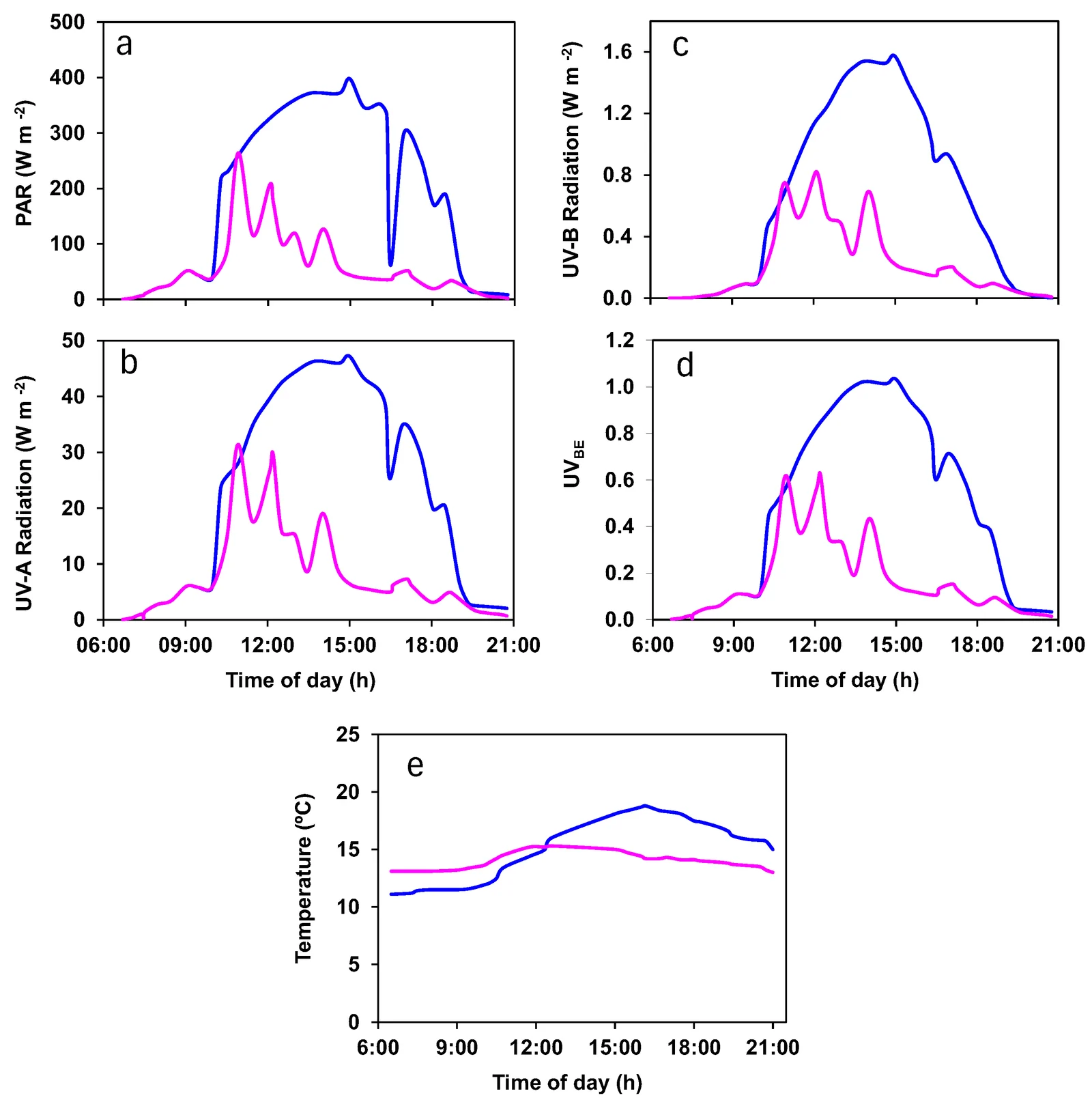
Diel changes in the environmental variables measured on two consecutive days (20–21 July) in the study site: (**a**), photosynthetically active radiation (PAR); (**b**), UV-A; (**c**), UV-B; (**d**), biologically effective UV irradiance (UV_BE_); and (**e**), water temperature. The first day of measurement is shown in blue and the second one in pink.

Changes in the environmental variables measured at the study site during the two sampling days (photosynthetic, UV-A, UV-B, and biologically effective UV (UV_BE_) irradiances, together with water temperature) are shown in Figure 1. The first sampling day was predominantly sunny, with only one marked cloudy episode in the afternoon. In contrast, the second day was very cloudy, especially from midday onwards, while sunshine alternated with cloud cover only during the morning. Maximum PAR reached approximately 398 W m^−2^ on the first day but only 264 W m^−2^ on the second day. Maximum UV-B irradiance was 1.57 and 0.82 W m^−2^ on the first and second days, respectively. Water temperature ranged from 11.1 to 18.8 °C on the first day and from 13.0 to 15.3 °C on the second day.

All radiation variables varied concomitantly on both sampling days. On the first day, irradiance values reached maxima around solar noon and minima at both dawn and dusk. On the second day, irradiances remained very low for most of the day, except during the approximately 4-h period preceding solar noon, when several peaks and troughs associated with changing cloud cover were recorded. Water temperature followed a pattern similar to that of irradiance, although with a certain delay, particularly on the first day. During this day, temperatures were low in the morning, increased until mid-afternoon, and then progressively decreased. Temperature fluctuations were less pronounced on the second day, consistent with the smaller changes observed in irradiance. Maximum temperatures occurred during the morning, whereas lower values were recorded throughout the remainder of the day. All irradiance variables (PAR, UV-A, UV-B, and UV_BE_) were highly intercorrelated (*p* < 0.001; Supplementary Table S1). Water temperature was significantly correlated with UV-A, UV-B, and UV_BE_ irradiances (*p* < 0.05), and with the PAR, UV-A, UV-B, and UV_BE_ accumulated doses (*p* < 0.01; Supplementary Table S1).

### 2.2. Diel Variation in Chlorophyll Fluorescence

**Figure 2:**
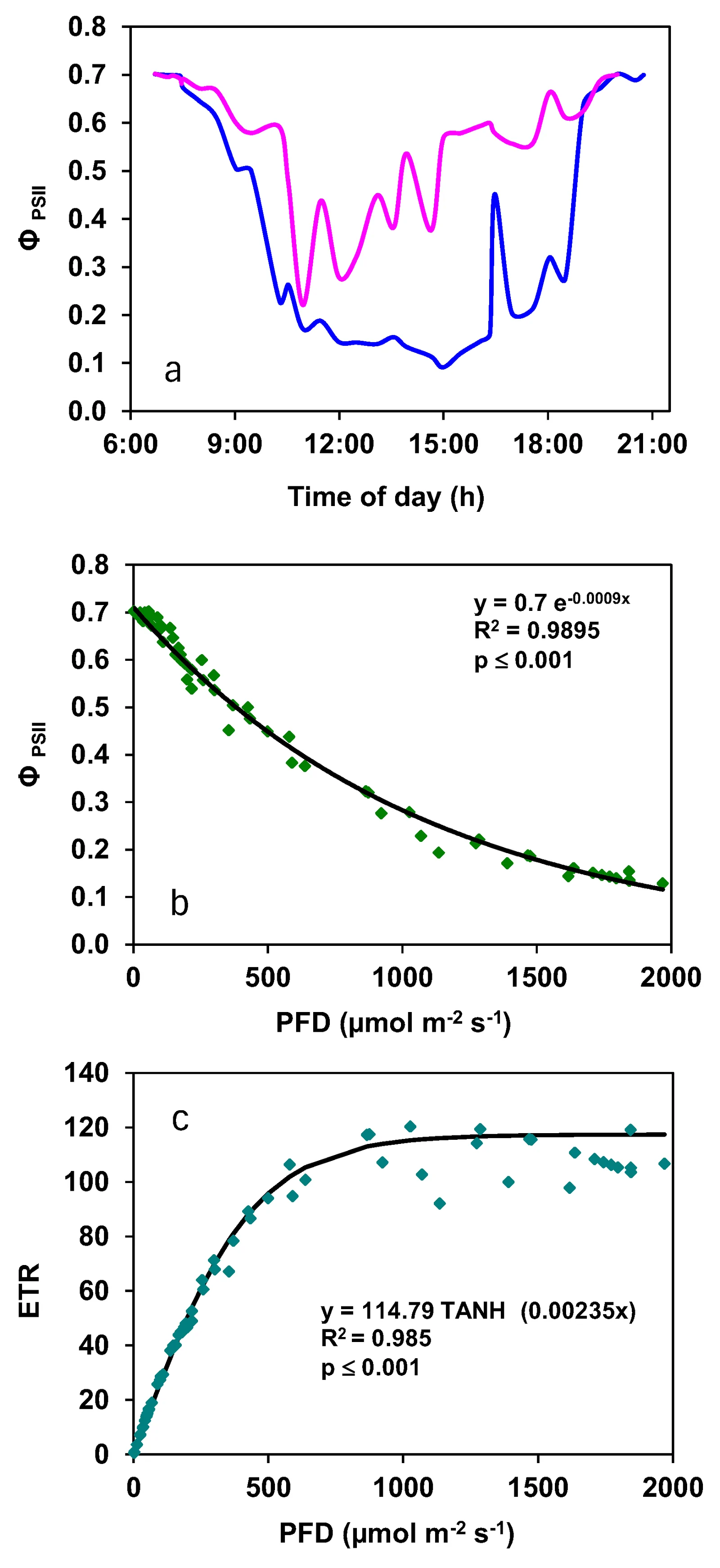
Diel changes in the effective photochemical quantum yield of photosystem II (Φ_PSII_) of *Jungermannia eucordifolia*, measured continuously on two consecutive days ((**a**); first day in blue and second day in pink), together with the fitted curves Φ_PSII_ vs. photon flux density (PFD, 400–700 nm) (**b**), and the apparent electron transport rate through photosystem II (ETR) vs. PFD (**c**). For fitted curves, the respective equations and determination coefficients are shown.

On both sampling days, variation in the effective photochemical quantum yield of photosystem II (Φ_PSII_) appeared to be inversely related to the changes in irradiances. On the first day, Φ_PSII_ showed low values during the central hours of the day (between 11:00 and 16:00 h) and high values in the morning and evening. On the second day, it exhibited several peaks and troughs around solar noon and, again, high values during the morning and evening (Figure 2). Φ_PSII_ values were generally higher on the second than on the first sampling day, except during the early morning and late evening hours, when values were similar on both days. When all Φ_PSII_, apparent electron transport rate through photosystem II (ETR(II), hereafter ETR), and photon flux density (PFD, 400–700 nm) values from both sampling days were considered together, the relationship between Φ_PSII_ and PFD was best described by a highly significant negative exponential function, whereas the relationship between ETR and PFD was best fitted by a highly significantly hyperbolic tangent function (Figure 2).

**Figure 3:**
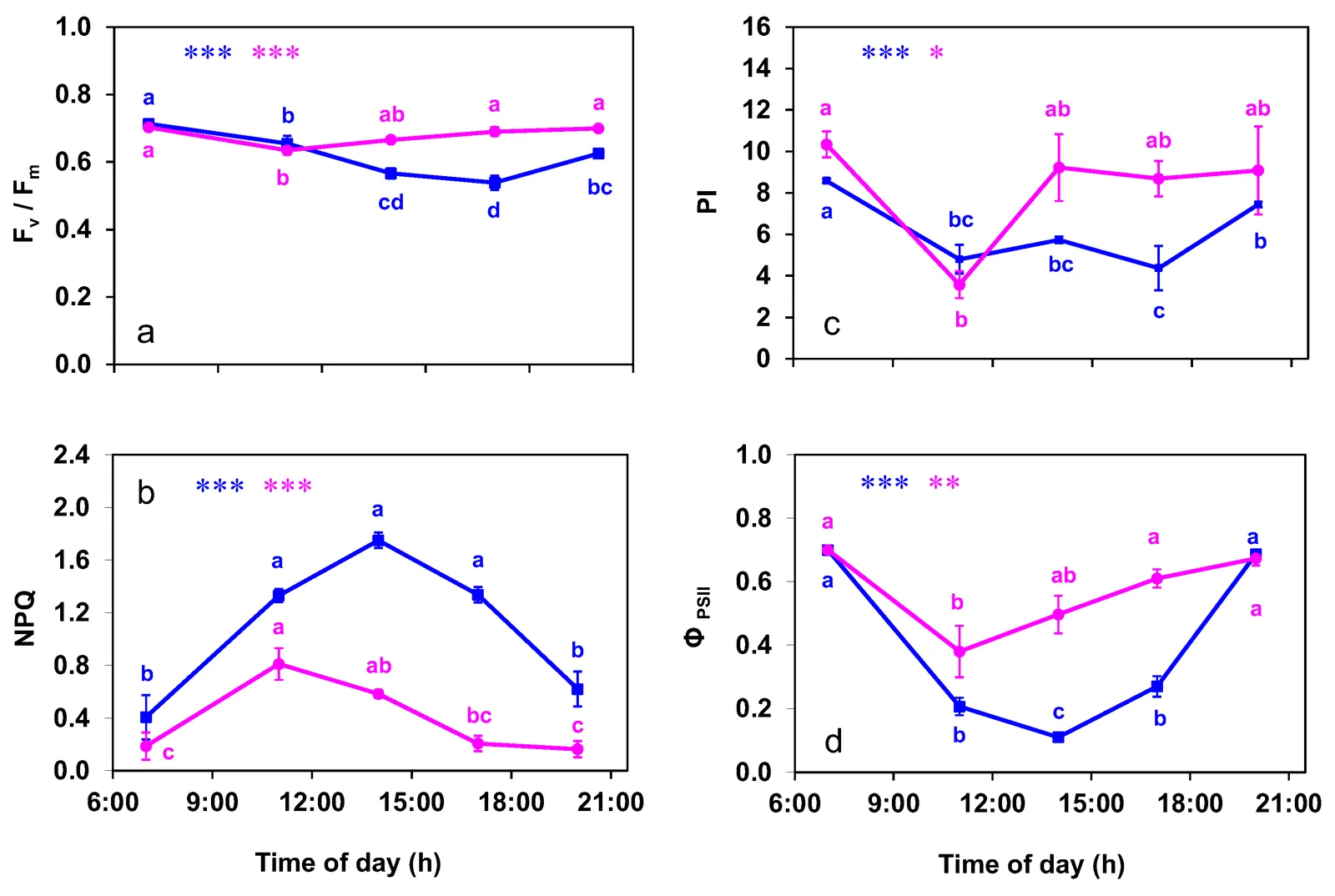
Diel changes in four variables of chlorophyll fluorescence ((**a**), F_v_/F_m_, maximum photochemical quantum yield of photosystem II (PSII); (**b**), NPQ, non-photochemical quenching; (**c**), PI, performance index; and (**d**), Φ_PSII_, effective PSII photochemical quantum yield) of *Jungermannia eucordifolia*, measured in five different periods of the day on the two consecutive measurement days (first day in blue and second day in pink). Means ± standard error (SE) are shown. For each variable, the overall effect of the period of the day is shown for each measurement day (first day in blue and second day in pink). This effect was tested by either a one-way ANOVA or a Kruskal–Wallis test (*** *p* < 0.001, ** *p* < 0.01, * *p* < 0.05). For each measurement day, different letters mean significant differences at least at *p* < 0.05.

Diel changes in PI, derived from the OJIP fluorescence transients, are shown in Figure 3. On the first sampling day, PI reached its highest values before dawn, then declined significantly to a plateau that persisted throughout most of the day, and finally increased significantly before dusk, although without reaching the pre-dawn maximum values. On the second day, after the high values recorded before dawn, PI decreased significantly during the morning and subsequently increased, fully recovering the initial values, which were then maintained throughout the remainder of the day.

Diel changes in F_v_/F_m_ and NPQ, together with those of Φ_PSII_ when only the five discrete measurements per day were considered, were more pronounced on the first than on the second sampling day (Figure 3). On the first day, F_v_/F_m_ followed a pattern broadly similar to that of PI. F_v_/F_m_ reached maximum values before dawn, then declined progressively and significantly until late afternoon, and finally increased significantly before dusk, although without fully recovering the pre-dawn values. Φ_PSII_ showed a similar pattern to F_v_/F_m_, although changes were more pronounced and clearly significant throughout the day. It decreased until solar noon and subsequently increased until the final measurement before dusk, completely recovering the values recorded before dawn. NPQ followed a clearly opposite trend to that of Φ_PSII_. On the second day, F_v_/F_m_ showed little diel variation, whereas Φ_PSII_ decreased significantly during the sunny morning period and subsequently increased significantly. Again, NPQ varied inversely with Φ_PSII_. Several chlorophyll fluorescence parameters were significantly correlated (Supplementary Table S1). F_v_/F_m_ and Φ_PSII_ were positively correlated with each other (*p* < 0.05) and negatively correlated with NPQ (at least *p* < 0.01). PI was positively correlated with Φ_PSII_ (*p* < 0.05) and negatively correlated with NPQ.

### 2.3. Diel Variation in UV-Absorbing Compounds

**Figure 4:**
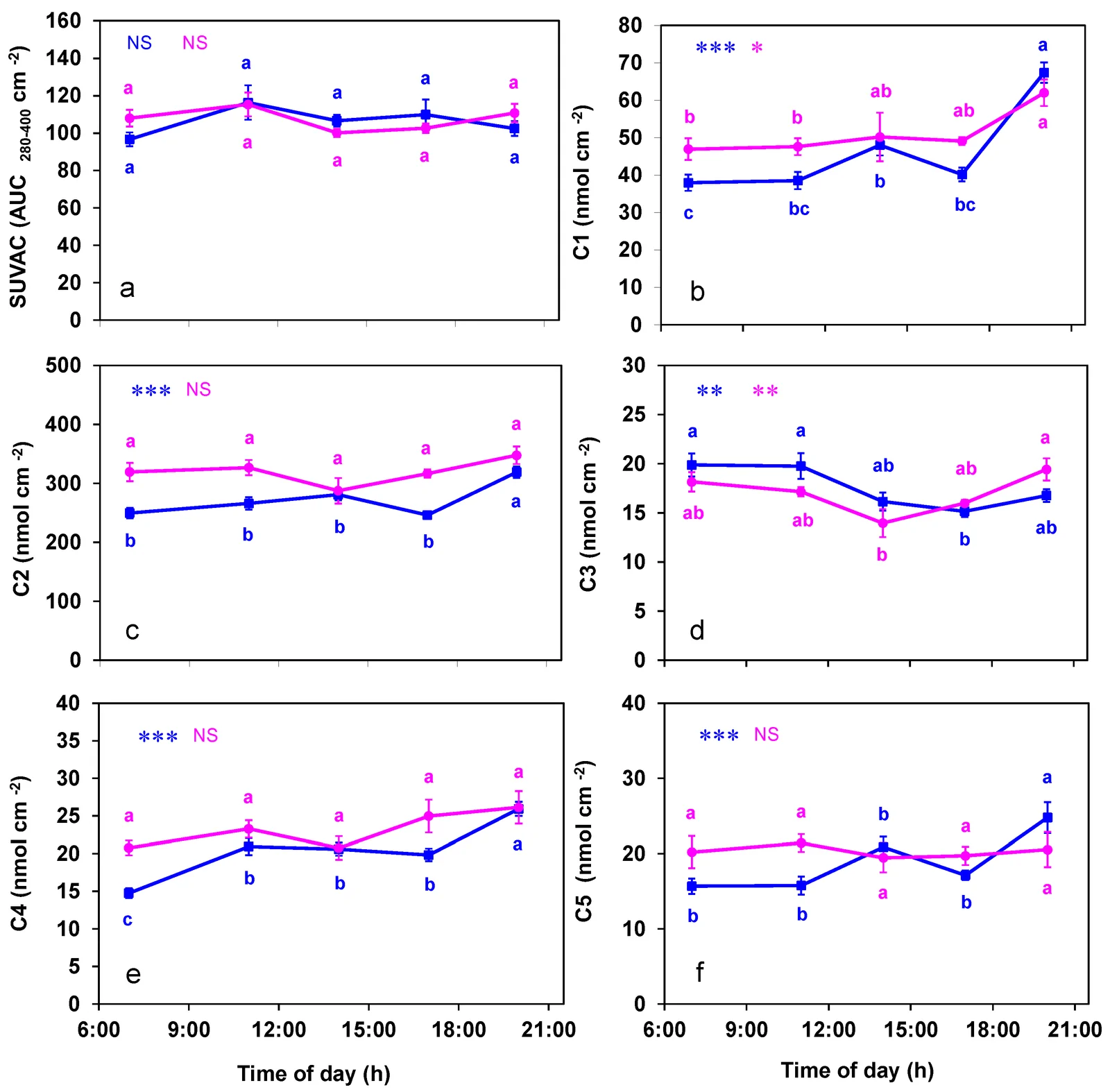
Diel changes in the bulk UV absorption capacity of methanol-soluble UV-absorbing compounds (SUVAC) (**a**) and the contents of the five individual UV-absorbing compounds from the soluble fraction (C1 to C5, (**b**–**f**), see names in the text) of *Jungermannia eucordifolia*, measured in five different periods of the day on the two consecutive measurement days (first day in blue and second day in pink). Means ± standard error (SE) are shown. For each variable, the overall effect of the period of the day is shown for each measurement day (first day in blue and second day in pink). This effect was tested by either a one-way ANOVA or a Kruskal–Wallis test (*** *p* < 0.001, ** *p* < 0.01, * *p* < 0.05, NS non-significant). For each measurement day, different letters mean significant differences at least at *p* < 0.05.

**Figure 5:**
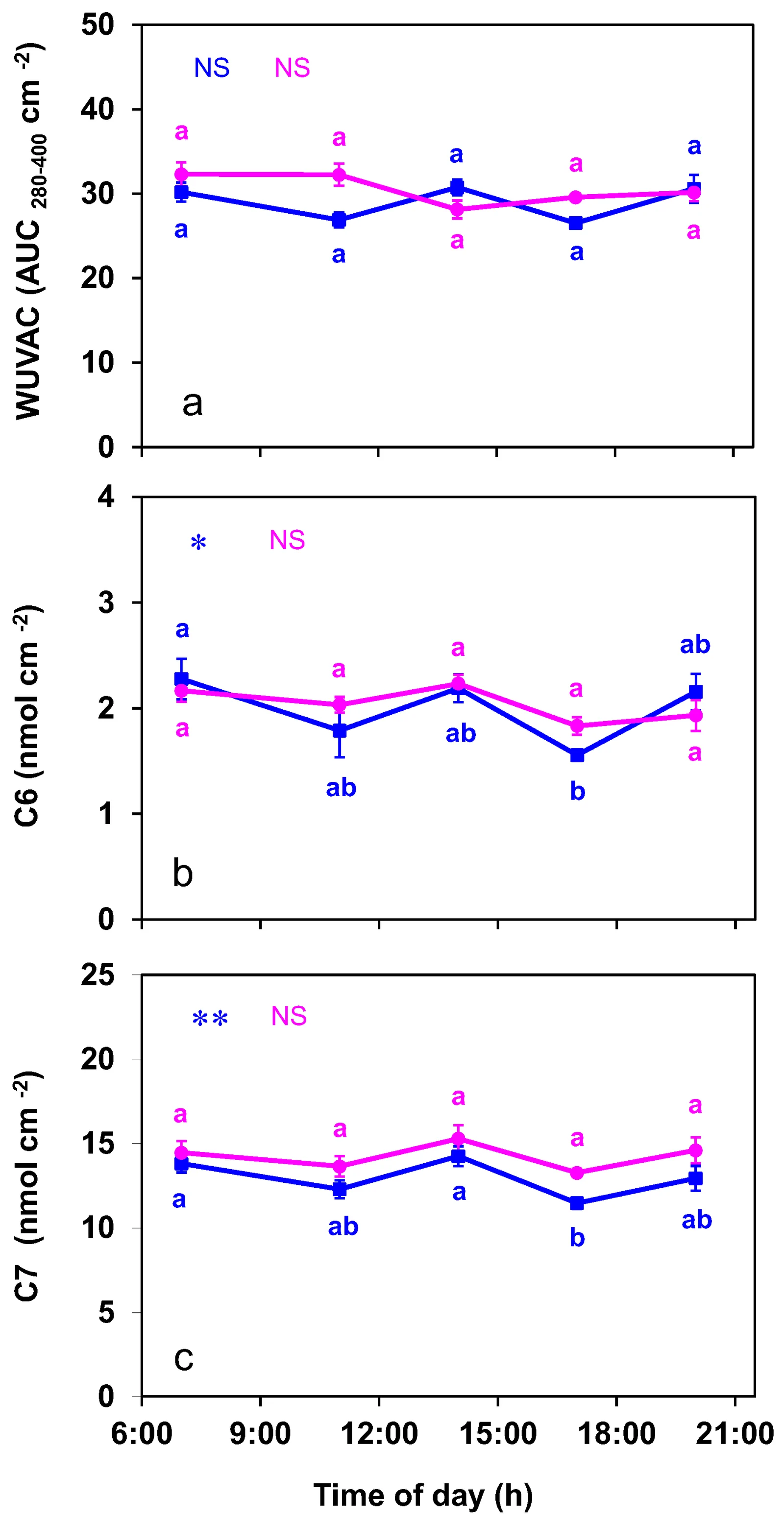
Diel changes in the bulk UV absorption capacity of methanol-insoluble cell wall-bound UV-absorbing compounds (WUVAC) (**a**) and the contents of the two individual UV-absorbing compounds from the insoluble fraction (C6, (**b**), and C7, (**c**), see names in the text) of *Jungermannia eucordifolia*, measured in five different periods of the day on the two consecutive measurement days (first day in blue and second day in pink). Means ± standard error (SE) are shown. For each variable, the overall effect of the period of the day is shown for each measurement day (first day in blue and second day in pink). This effect was tested by either a one-way ANOVA or a Kruskal–Wallis test (** *p* < 0.01, * *p* < 0.05, NS non-significant). For each measurement day, different letters mean significant differences at least at *p* < 0.05.

The spectrophotometric variables used to estimate the bulk UV absorption capacity of UV-absorbing compounds in the methanol-soluble and methanol-insoluble fractions (SUVAC and WUVAC, respectively) showed little or no diel variation on either sampling day (Figure 4 and Figure 5).

Regarding individual UV-absorbing compounds, seven compounds were identified: five in the methanol-soluble fraction and two in the methanol-insoluble fraction (Section 4.4). The soluble compounds were: *p*-coumaroylmalic acid (C1), caffeoylmalic acid (C2), feruloylmalic acid (C3), 5″-(7″,8″-dihydroxycoumaroyl)-2-caffeoylmalic acid (C4), and 5″-(7″,8″-dihydroxy-7-O-β-glucosyl-coumaroyl)-2-caffeoylmalic acid (C5). The insoluble compounds were *p*-coumaric acid (C6) and ferulic acid (C7). Figure 4 shows the temporal variation in the methanol-soluble compounds. C1, C2, and C5 remained relatively stable throughout the first sampling day but increased significantly before dusk. C3 decreased during the second half of the day, whereas C4 increased significantly in the morning, remained stable thereafter, and increased significantly again before dusk. On the second sampling day, changes were considerably smaller than those observed on the first day. C1 followed a pattern similar to that observed on the first day, with a significant increase before dusk, whereas C3 remained stable throughout the day until a significant increase occurred before dusk (as C1). No significant changes were detected for C2, C4, or C5. C1, C2, and C5 were positively intercorrelated (at least *p* < 0.05), C5 was positively correlated with C4 (*p* < 0.05), and C4 was positively correlated with C2 (*p* < 0.01; Supplementary Table S1).

The methanol-insoluble UV-absorbing compounds C6 and C7 (Figure 5) showed even smaller diel variations than the methanol-soluble compounds on both sampling days. On the first day, both compounds fluctuated throughout the day, whereas no significant changes were observed on the second day. C6 and C7 were positively correlated with each other (*p* < 0.05; Supplementary Table S1). No significant correlations were found between chlorophyll fluorescence parameters and variables related to UV-absorbing compounds (Supplementary Table S1).

### 2.4. Relationships Between Physiological and Environmental Variables

Several significant correlations were found between physiological and environmental variables (Supplementary Table S1). Irradiance variables were positively correlated with NPQ (*p* < 0.001) and negatively correlated with F_v_/F_m_ and Φ_PSII_ (at least *p* < 0.01). PI was negatively correlated only with PAR and UV-A irradiances (*p* < 0.05). Water temperature was negatively correlated with F_v_/F_m_ (*p* < 0.01) and C3 (*p* < 0.05), and positively correlated with NPQ (*p* < 0.05).

### 2.5. DNA Damage

**Figure 6:**
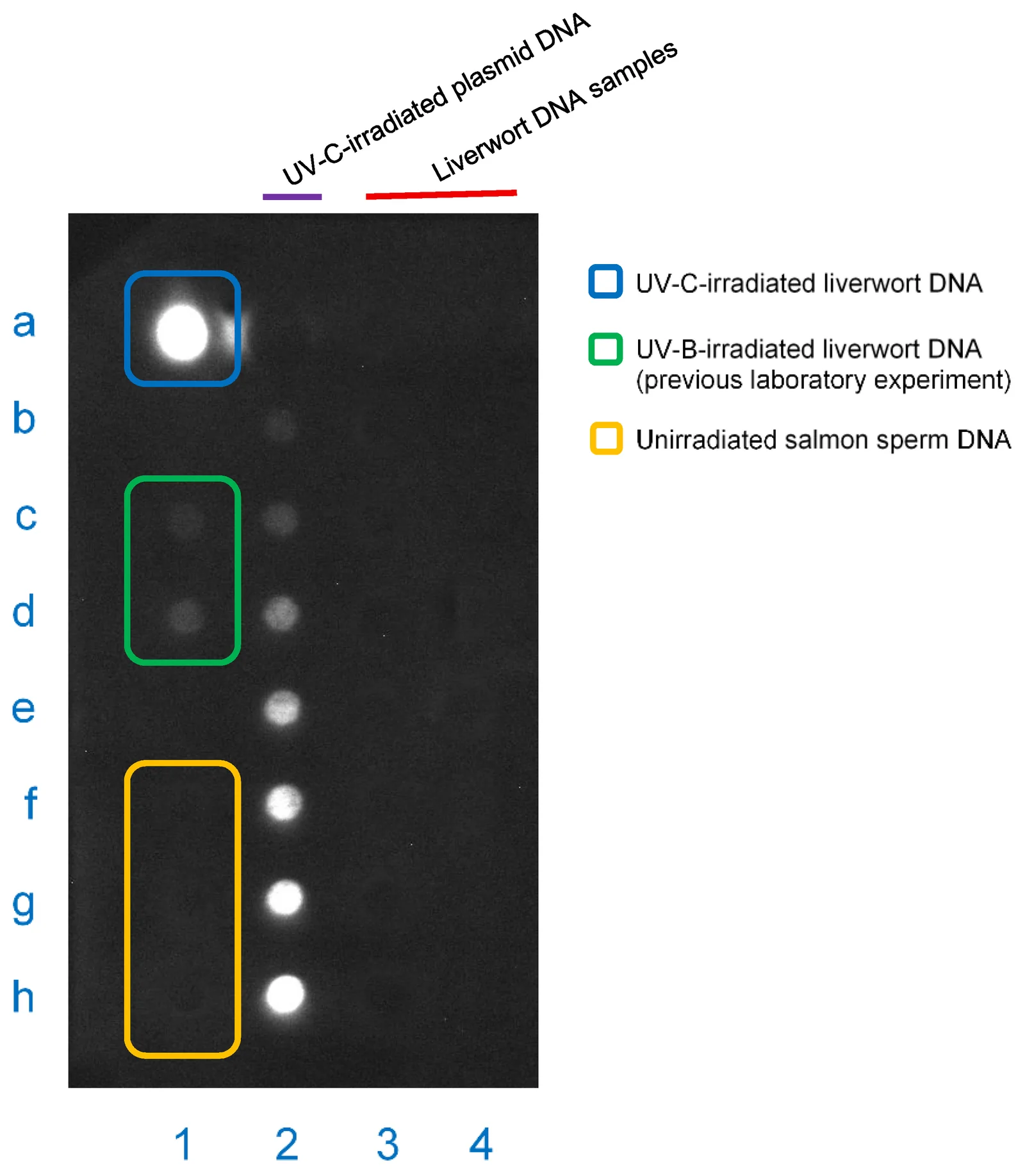
Results of the immunoassay performed to determine DNA damage (presence of thymine dimers). The dot blot shows: in column 1 (lanes f–h), unirradiated control samples of salmon sperm DNA, using 1, 2 or 3 µg DNA per dot, respectively; in column 2 (lanes a–h), increasing amounts (0, 0.5, 1, 2, 3, 4, 5 and 10 ng) of DNA from plasmid pBSK irradiated with UV-C (calibration column); in column 1 (lane a), a 250-ng DNA sample from *Jungermannia eucordifolia* irradiated with UV-C; in column 1 (lanes c and d), 1 and 2-µg DNA samples of the liverwort from a previous laboratory experiment in which they had been irradiated with UV-B and had shown damage; in columns 3 and 4 (lanes a–h), 1-µg DNA samples of the liverwort corresponding to at least one replicate for each sampling time on each sampling day.

Remarkably, no DNA damage was detected in any of the samples analyzed (Figure 6). Given this negative result, several precautions were taken to exclude potential methodological errors in the assessment of DNA damage in *J. eucordifolia* samples in this experiment. Simultaneously with the analysis of these samples, (1) DNA damage was assessed in UV-C-irradiated plasmid DNA as a standard calibration procedure; (2) unirradiated salmon sperm DNA was verified to be free of damage; and (3) DNA damage previously demonstrated in *J. eucordifolia* exposed to both UV-C and UV-B under laboratory conditions [19] was used as a positive control. These additional analyses confirmed the absence of DNA damage in the liverwort samples collected during the present field experiment.

## 3. Discussion

The two sampling days differed markedly in their meteorological conditions, with the first day being predominantly sunny and the second largely overcast. This natural contrast provided an excellent opportunity to compare the physiological responses of *J. eucordifolia* under contrasting environmental scenarios. Such rapid weather fluctuations, even during mid-summer, are relatively common in mountain regions [20]. Radiation variables exhibited stronger and more dynamic changes than water temperature (Figure 1). This was expected given that solar radiation inherently undergoes pronounced daily fluctuations that are further modified by cloud cover, whereas water has a high thermal buffering capacity. As a result, water temperature was more predictable than radiation and responded with a certain delay to changes in solar radiation. Consequently, water temperature was more strongly correlated with accumulated radiation doses than with instantaneous irradiances (Supplementary Table S1). Overall, both radiation and water temperature varied substantially between the two sampling days and, particularly in the case of radiation, within each day. This environmental variability provided a suitable framework for evaluating the short-term responsiveness of the different variables measured in the liverwort.

Chlorophyll fluorescence parameters (Φ_PSII_, F_v_/F_m_, NPQ, PI) showed consistent diel patterns, particularly on the first sampling day, when fluctuations in irradiance were more pronounced. The simultaneous variation of all radiation wavebands precluded a definitive attribution of the observed responses to a specific spectral region. However, previous manipulative studies conducted on bryophytes [1,21] and tracheophytes [17] under both field and laboratory conditions suggest that PAR was probably the main driver of these changes, exerting a stronger influence than UV irradiance on photosynthetic performance. The decrease in Φ_PSII_ observed during periods of high PAR irradiance, approximately between 10:00 and 18:00 h on the first day and during transient sunny episodes on the second day, resulted in a significant negative exponential relationship between Φ_PSII_ and PFD (Figure 2). The reduction in Φ_PSII_ was most likely associated with increased thermal dissipation of excess absorbed energy. This response would be largely mediated by the increased activity of the xanthophyll cycle, as indicated by the concomitant increase in NPQ, which functions as an important photoprotective mechanism preventing overexcitation of PSII under high irradiance [22]. This interpretation is strongly supported by the significant negative correlation between NPQ and Φ_PSII_, as well as by their contrasting diel patterns (Figure 3). Nevertheless, the increase in NPQ observed around midday may also reflect the contribution of alternative non-photochemical energy dissipation pathways [13]. The transient decreases in Φ_PSII_ observed throughout the day represent a common feature of photosynthesis in natural environments and are generally interpreted as a down-regulation that maintains a balance between light-driven electron flow and requirements for reducing power [21,22]. Likewise, reduced photosynthetic efficiency in mosses exposed to high irradiance under natural conditions has been interpreted as a reversible photoprotective state rather than evidence of permanent damage, with thermal energy dissipation mediated by xanthophyll-cycle pigments playing a central role [23].

F_v_/F_m_ is a widely accepted indicator of photoinhibition, and reductions in this parameter are generally associated with impairment of photosystem II (PSII) function [13]. Therefore, the decrease in the afternoon followed by an incomplete recovery before dusk suggests the occurrence of dynamic photoinhibition, consistent with previous observations under laboratory conditions [21]. Although high PAR was probably the main factor responsible for this photoinhibitory response, UV radiation may also have contributed, as decreases in F_v_/F_m_ are commonly observed in bryophytes exposed to enhanced UV-B radiation [1,9,24]. PI followed a temporal pattern broadly similar to that of F_v_/F_m_, and therefore provided comparable information on photosynthesis performance. However, PI exhibited larger diel fluctuations, suggesting that it may be a more sensitive indicator of radiation-induced stress, as has also been reported for other environmental stressors [25].

Overall, the diel patterns of chlorophyll fluorescence observed in the present study are largely consistent with those previously described in other bryophytes [8,9,10,11] and tracheophytes [26]. These findings indicate that *J. eucordifolia* is well adapted to its natural niche and that the environmental conditions experienced during the study induced a dynamic photoinhibition rather than chronic physiological damage.

In *J. eucordifolia*, ETR increased with increasing PFD until reaching saturation, and the relationship was accurately described by a hyperbolic tangent function (Figure 2). Saturation occurred at relatively high PFD levels, consistent with previous laboratory observations [21], although the saturation irradiance observed under field conditions was approximately twice that reported in controlled experiments. This observation was accompanied by higher Φ_PSII_ values under natural conditions at comparable PFD levels. Laboratory experiments reported Φ_PSII_ values of approximately 0.20 at 500 µmol m^−2^ s^−1^ PFD [21], whereas field measurements in the present study reached values close to 0.45 at similar PFD.

Interestingly, *J. eucordifolia* showed relatively low NPQ values compared with those reported for other bryophytes [27,28], a characteristic generally associated with shade-adapted species. However, this interpretation appears inconsistent with the relatively high PFD levels required for ETR saturation in this liverwort. Several explanations may account for this apparent discrepancy. First, methodological differences exist among studies, particularly because the measurements reported by Marschall & Proctor [27] and Proctor & Smirnoff [28] were conducted over short periods under controlled laboratory conditions. Second, the *J. eucordifolia* populations used in our study were permanently submerged, avoiding the concurrence of high radiation stress and hydric stress; this might reduce the need for strong photoprotection, and thus NPQ could be maintained low.

The bulk UV absorbance of both the methanol-soluble (SUVAC) and the methanol-insoluble (mainly cell wall-bound compounds, WUVAC) fractions did not show clear diel changes on either sampling day (Figure 4 and Figure 5). Regarding the individual compounds, diel variations were somewhat more pronounced on the first than on the second sampling day (Figure 4 and Figure 5), suggesting that these changes may have been induced, at least to some extent, by the higher radiation levels recorded on the first day. However, most individual UV-absorbing compounds did not exhibit clear diel patterns, indicating that their response to the increase in radiation during the first sampling day (approximately between 10:00 and 18:00 h) was weak or absent. Overall, most UV-absorbing variables, particularly the bulk UV absorbance of SUVAC and WUVAC, did not show consistent diel patterns, although some individual compounds exhibited significant fluctuations at specific times of the day. Surprisingly, the most frequent change observed among the individual UV-absorbing compounds in the present study was a significant increase towards the end of the day. This pattern was observed for C1, C2, C4, and C5 on the first sampling day, and for C1 and C3 on the second day. These findings contrasted strongly with the significant diel changes in both bulk UV absorbances, as well as in compounds C6 and C7, previously reported for *J. eucordifolia* under laboratory conditions [21]. In that study, these variables increased at the end of a period of high PAR and UV applied within an artificial day, and the responses were mainly attributed to enhanced UV-B. In addition, several other compounds increased in the same species in response to enhanced UV-B under different experimental conditions. Among the UV-absorbing compounds analysed, C1 consistently showed the strongest responses to enhanced UV-B under both laboratory [21] and field [1,2] conditions, suggesting that it may be the most reliable UV-B biomarker. Indeed, under field conditions, C1 was strongly correlated with PAR, UV-A, and UV-B radiation throughout a three-year seasonal cycle [2], while laboratory studies demonstrated that C1 content increased specifically in response to enhanced UV-B during cultivation periods ranging from 1 to 15 days [21]. Overall, the following recommendations can be suggested to design an effective UV-B biomonitoring program based on the C1 content in *J. eucordifolia*: (1) because this liverwort is aquatic, sampling should be based on permanently submerged populations, thereby avoiding the interference caused by the desiccation–rehydration cycles typical of bryophytes; (2) the diel behaviour of C1 should be considered to determine the most appropriate sampling time; in this regard, the relatively stable C1 levels observed throughout most of the day suggest that sampling could be performed at almost any time of day, although the final hours of daylight should preferably be avoided because of the unexplained increase in C1 content observed during this period; and (3) there is a need for sampling periods longer than a single day, because C1 did not show a clear diel response to UV-B under field conditions and its response appeared to occur more slowly than under laboratory conditions.

Rapid changes in the bulk UV absorbance of SUVAC of bryophytes have previously been reported under field conditions in response to increases in UV-B radiation caused either by Antarctic ozone depletion [5,6,7] or by artificial UV-B supplementation using lamps [3]. However, no clear daily pattern in UV-absorbing compounds was detected in those studies, nor in a study conducted on a thalloid liverwort [9], when plants were exposed to ambient solar radiation, as in the present work. Thus, diel changes in UV-absorbing compounds may be more difficult to detect under natural solar radiation than under experimentally enhanced UV-B conditions in either field or laboratory studies. In *J. eucordifolia*, the lack of clear diel variation could be attributed to the fact that submerged aquatic bryophytes may not require particularly high contents of protecting UV-absorbing compounds, because the water column itself provides a certain degree of protection against UV radiation [29]. However, this explanation is unlikely in the present study because the stream water was oligotrophic, contained little organic matter, and the plants were submerged by only a few centimetres owing to the relatively low summer discharge. Under these conditions, UV-B attenuation along the water column is negligible [29]. Several alternative explanations may account for the absence of clear diel changes in UV-absorbing compounds. First, the effects of UV radiation may have been masked by other environmental factors operating simultaneously [4]. Second, multiple physiological processes may occur concurrently within the plant, including the interconversion of UV-absorbing compounds that share common biosynthetic pathways, the redistribution of compounds between vacuoles and cell walls, and changes in substrate availability or enzymatic activity involved in phenolic biosynthesis in response to environmental or endogenous factors [18]. Third, permanently submerged aquatic bryophytes may experience less pronounced physiological fluctuations than terrestrial bryophytes because the buffering capacity of water reduces the impact of environmental variability, particularly temperature fluctuations [2], leading to a relatively stable physiological state of the plant over time. Fourth, metabolite responses can be triggered by relatively low UV-B levels, due to the specific action of the UV-B photoreceptor UVR8 [4]. Finally, the coexistence of constitutive UV-protection mechanisms and slowly adjustable protective strategies may further dampen short-term diel variability under natural conditions [23]. Although clear diel changes in UV-absorbing compounds were not detected in the present study, substantial variation may occur over longer temporal scales, such as seasonal cycles [2].

Notably, no significant correlations were found between chlorophyll fluorescence parameters and UV-absorbing compounds (Supplementary Table S1). This finding suggests that photosynthetic performance and UV protection may be regulated by different combinations of environmental factors. Furthermore, these processes may operate over different temporal scales, with chlorophyll fluorescence-related processes showing greater short-term dynamics than the synthesis and accumulation of UV-absorbing compounds.

DNA damage was not detected in any of the samples analyzed. This contrasted with the results obtained in a laboratory diel cycle under artificially enhanced UV-B, where DNA damage increased at the end of a period of high PAR and UV exposure, although it was completely repaired after UV irradiation ceased [19]. Several studies have demonstrated DNA damage in bryophytes exposed to artificially enhanced UV-B under both laboratory and field conditions [1,10,11,30], which is consistent with the well-established sensitivity of DNA to UV-B [31]. In contrast, and in agreement with our findings, exposure to ambient UV-B levels under natural field conditions did not result in detectable DNA damage [1,2,10,11,32]. This may reflect the effectiveness of the protective and repair mechanisms operating in bryophytes under natural conditions. The only study reporting DNA damage in bryophytes exposed to ambient UV-B radiation was conducted in Antarctica [33], where low temperatures probably limited the efficiency of DNA repair processes.

All radiation variables (PAR, UV-A, UV-B, and UV_BE_) were highly intercorrelated (Supplementary Table S1), making it impossible to fully discriminate their individual effects. Such multicollinearity was expected because PAR, UV-A, and UV-B irradiances and doses are usually closely associated under natural conditions [2]. This represents an inherent limitation of observational field studies and precludes a rigorous statistical separation of the effects of the different spectral regions. Water temperature was also correlated with several radiation variables (Supplementary Table S1), further complicating the identification of the individual contributions of each environmental factor. Nevertheless, based on previous laboratory studies [19,21], it is possible to infer the likely environmental drivers of the observed physiological responses. In particular, the dynamic changes observed in chlorophyll fluorescence parameters were most likely associated with variations in the PAR environment [21]. Nevertheless, the present study identifies several avenues for future research that could contribute to a better understanding of the field responses of aquatic bryophytes to environmental factors, particularly to UV radiation. In this regard, manipulative experiments employing lamps and/or spectral filters are needed to disentangle the effects of different wavelength regions. Such studies have rarely been conducted in stream bryophytes [34], largely because of the logistical difficulties associated with working in highly dynamic lotic ecosystems.

Overall, the integrative approach applied in the present study, in which diel changes in photosynthetic performance, UV-absorbing compounds, and DNA integrity were simultaneously assessed in the aquatic liverwort *J. eucordifolia* under field conditions, revealed marked differences in the temporal behaviour of the physiological responses analysed. Specifically, chlorophyll fluorescence parameters related to photosynthetic performance (Φ_PSII_, F_v_/F_m_, and PI) and photoprotection against excess radiation (NPQ) were more responsive to short-term environmental fluctuations than variables associated with UV photoprotection through UV-absorbing compounds. Chlorophyll fluorescence parameters showed clear and consistent diel patterns, whereas UV-absorbing compounds generally did not, although some individual compounds exhibited significant diel fluctuations. No DNA damage was detected at any sampling time. Therefore, *J. eucordifolia* appears to be well adapted to ambient levels of PAR and UV radiation without the need to invest substantial additional resources in the rapid synthesis of protective compounds in response to diel fluctuations in irradiance. These findings support the current view of UV radiation as a specific regulator rather than a general stressor in plants [1].

In addition, comparison of the diel physiological responses observed under field and laboratory conditions indicates that similarities and differences depend on the variable considered. Chlorophyll fluorescence parameters, including Φ_PSII_, F_v_/F_m_, NPQ, and PI, exhibited broadly similar diel patterns under both field and laboratory conditions [9,11,12,21], as have also been reported for tracheophytes [15]. In contrast, UV-absorbing compounds appear to be more readily induced under laboratory conditions, possibly because of the higher UV irradiances and/or doses typically applied in such studies [21], although exceptions have been reported [9]. Similarly, DNA damage is more readily detected under laboratory conditions [10,11,19], probably for the same reasons and because DNA repair processes may be more effective under field conditions. It could be hypothesized that this contrasting field-*versus*-laboratory behaviour of UV-absorbing compounds and DNA damage responses is partially related to differences in UVR8-mediated signalling [4]. However, further studies are required to evaluate this possibility. These results highlight the importance of conducting field experiments to achieve a realistic understanding of plant behaviour under natural conditions.

Finally, aquatic bryophytes such as *J. eucordifolia* have been proposed as promising biomonitors of UV-B changes in the biosphere, including those associated with anthropogenic degradation of stratospheric ozone [1,2]. In this context, the present field study provides valuable information for evaluating the potential usefulness of specific physiological variables in UV biomonitoring. This may contribute to improving methodological approaches in this field of research and may ultimately be relevant to programs associated with the Montreal Protocol [35].

## 4. Materials and Methods

### 4.1. Plant Material and Experimental Site

Samples of the leafy aquatic liverwort *Jungermannia eucordifolia* Schljakov were monitored in situ over two consecutive days (20–21 July 2011) in the Lavieja stream (1280 m a.s.l., 42°03′00″ N, 02°35′30″ W), in La Rioja (northern Spain). The samples were submerged and exposed to full sunlight. The vegetation surrounding the sampling site consisted of open shrubland dominated by *Cytisus scoparius* L., with scattered beech trees (*Fagus sylvatica* L.).

### 4.2. Environmental Data

Water temperature was measured in situ every 30 min throughout the sampling period using a Testo 106 thermometer (Testo AG, Lenzkirch, Germany). Temperature measurements were taken at the depth where *J. eucordifolia* was growing (approximately 20 cm). Photosynthetic (PAR), UV-A and UV-B irradiances were continuously monitored using a quantum sensor (LI-190SA, LI-COR, Lincoln, NE, USA) and a spectroradiometer (Macam SR9910, Macam Photometrics Ltd., Livingstone, Scotland). Biologically effective UV irradiance (UV_BE_) was calculated using the action spectrum of Flint & Caldwell [36]. Accumulated doses (kJ m^−2^) of PAR, UV-A, UV-B, and UV_BE_ were also calculated at different times throughout the day.

### 4.3. Chlorophyll Fluorescence

Chlorophyll *a* fluorescence was measured in situ. Minimal and maximal fluorescence (F_0_ and F_m_) was determined with a MINI-PAM portable fluorometer (Walz Instruments, Effeltrich, Germany) using the saturation pulse method on samples that had been dark-adapted for 20 min. F_0_ was recorded using a modulated red measuring light (650 nm) of 0.03 μmol m^−2^ s^−1^ photon flux density (PFD, 400–700 nm), whereas F_m_ was induced by a saturating pulse of 12,000 μmol m^−2^ s^−1^ PFD with a duration of 0.8 s. The maximum photochemical quantum yield of photosystem II (PSII) was calculated as F_v_/F_m_, where F_v_ = F_m_ − F_0_. The effective PSII photochemical quantum yield (Φ_PSII_) was measured in samples exposed to ambient solar radiation and calculated as (F_m_′ − F_t_)/F_m_′, where F_m_′ represents the maximum fluorescence in the light-adapted state and F_t_ is the steady-state fluorescence. For each determination of Φ_PSII_, F_t_ was recorded immediately before application of a single saturating pulse, during which F_m_′ was obtained. F_m_′ was determined using the same saturating pulse settings described above for dark-adapted samples (12,000 μmol m^−2^ s^−1^ PFD, 0.8 s). During each Φ_PSII_ measurement, the incident PFD reaching the sample was recorded using the sensor incorporated into the MINI-PAM leaf clip. The apparent electron transport rate through PSII (ETR(II), usually referred to as ETR) was calculated as: ETR = Φ_PSII_ × PFD × 0.5 × 0.84. Quenching due to non-photochemical dissipation of absorbed light energy (NPQ) was calculated as (F_m_ − F_m_′)/F_m_′. Since measurements were performed under natural field conditions, samples remained exposed to the ambient irradiance prevailing at each sampling time until fluorescence measurements were taken and were therefore assessed in their naturally light-acclimated state. Accordingly, no artificial actinic light was applied. The relationship between Φ_PSII_ and PFD throughout the day was fitted to a negative exponential function, whereas the relationship between ETR and PFD was fitted to the hyperbolic tangent equation of Jassby and Platt: Y(X) = Ymax tanh (αX/Ymax), where Ymax is the maximum value of Y and α is the initial slope [37].

In addition, fast chlorophyll fluorescence induction kinetics (OJIP transients) were recorded using an AquaPen-P AP-P 100 fluorometer (Photon Systems Instruments, Brno, Czech Republic) and analyzed according to the JIP-test approach [14,15]. Briefly, chlorophyll fluorescence transients were induced by a saturating PFD of 3000 µmol m^−2^ s^−1^ and relative variable fluorescence was recorded at different time intervals between 10 µs and 2 s. Relative variable fluorescence was calculated as V_t_ = (F_t_ − F_0_)/(F_m_ − F_0_). The cardinal points of the OJIP curve and the derived parameters were calculated using Fluorpen 2.0 software. From these data, the Performance Index (PI) was obtained as a multiparametric expression integrating the three independent steps contributing to photosynthesis: light energy absorption, excitation energy trapping, and conversion of excitation energy into electron transport.

### 4.4. UV-Absorbing Compounds

UV-absorbing compounds were analyzed by both spectrophotometry and HPLC [21]. Samples were collected in the field, and the surface area of the prostrate apices was measured in situ using a LI-COR LI-3000 area meter (Lincoln, NE, USA). Samples were immediately frozen in liquid N_2_ and transported to the laboratory. The bulk UV absorbance of both methanol-soluble compounds (SUVAC), presumed to be mainly vacuolar, and methanol-insoluble compounds (WUVAC), was determined spectrophotometrically (Perkin–Elmer λ35 spectrophotometer, Perkin–Elmer, Wilton, CT, USA). Briefly, extraction was performed in acidified methanol. Following centrifugation, the supernatant and pellet were considered the sources of SUVAC and WUVAC, respectively [16]. In the supernatant, the bulk UV absorption capacity of SUVAC was measured as the area under the absorbance curve between 280 and 400 nm (AUC_280–400_) per unit surface area. The remaining pellet was hydrolyzed with NaOH, acidified with HCl, and extracted three times with ethyl acetate. Following evaporation, the residue was dissolved in methanol, and the bulk UV absorption capacity of WUVAC was measured spectrophotometrically as described above for SUVAC. Presumably, the methanol-insoluble fraction (WUVAC) mainly represents cell wall-bound compounds [16] because cell walls would be the prevailing cell debris in the pellet, although the precise chemical composition of this fraction might be more heterogeneous and remains unresolved.

Seven hydroxycinnamic acid derivatives were analysed by HPLC (Agilent HP1100 HPLC system, Agilent Technologies, Palo Alto, CA, USA), five in the methanol-soluble fraction and two in the methanol-insoluble fraction. The soluble compounds were *p*-coumaroylmalic acid, caffeoylmalic acid (or phaselic acid), feruloylmalic acid, 5″-(7″,8″-dihydroxycoumaroyl)-2-caffeoylmalic acid, and 5″-(7″,8″-dihydroxy-7-O-β-glucosyl-coumaroyl)-2-caffeoylmalic acid, hereafter referred to as C1 to C5, respectively. The insoluble compounds were *p*-coumaric and ferulic acids, hereafter referred to as C6 and C7, respectively. The extraction, purification and structural identification of these phenolic compounds had previously been performed for this species using chromatographic and spectroscopic techniques (UV, IR, NMR and MS) [38]. In the present study, these previously characterized compounds were quantified by HPLC-DAD. Quantification was based on integration of chromatographic peak areas recorded at 324 nm, the wavelength selected for the detection of hydroxycinnamic acid derivatives. Peak areas were converted into compound contents using calibration curves constructed with the corresponding standards [38]. Results were expressed on a surface-area basis (nmol cm^−2^).

### 4.5. DNA Damage

DNA damage was evaluated by detecting thymine dimers [39]. Samples were collected, immediately frozen in liquid N_2_, and transported to the laboratory. DNA damage calibration was performed using UV-C-irradiated DNA from plasmid pBSK. In addition, we verified that non-irradiated salmon sperm DNA showed no detectable DNA damage and that DNA from *J. eucordifolia* exposed to UV-B radiation in a previous laboratory experiment [19] exhibited detectable DNA damage.

### 4.6. Diel Sampling and Replicates

All the variables (F_v_/F_m_, Φ_PSII_, NPQ, OJIP curves, PI, SUVAC, WUVAC, the contents of C1 to C7, and DNA damage) were measured at five sampling times on each of the two consecutive days: before dawn (darkness) and approximately at 11:00, 14:00, 17:00, and 20:00 h (local time, 2-h ahead of solar time). In addition, Φ_PSII_ was measured every 30 min from 06:30 to 20:30 to better assess its dynamic changes, to calculate ETR, and to obtain the regressions between Φ_PSII_ and PFD and between ETR and PFD.

Chlorophyll fluorescence parameters (including PI) were measured in three replicate samples. In addition, Φ_PSII_ was continuously monitored in one replicate. SUVAC, WUVAC, and C1-C7 contents were determined in five replicates, whereas DNA damage was assessed in three replicates.

### 4.7. Statistical Analysis

The overall effect of sampling time on each physiological variable was evaluated using one-way analysis of variance (ANOVA), after verifying that the assumptions of normality (Shapiro–Wilk’s test) and homoscedasticity (Levene’s test) were satisfied. When significant differences were detected, means were compared using Tukey’s post-hoc test. If the assumptions for ANOVA were not met, Kruskal–Wallis tests were performed. When significant differences were detected, pairwise comparisons were conducted using Mann–Whitney’s tests. Pearson’s correlation coefficients were calculated between all physiological and environmental variables (Supplementary Table S1). All statistical analyses were performed using SPSS 24.0 for Windows (SPSS Inc., Chicago, IL, USA) [40].

## Data Availability

The original data presented in the study are openly available in Zenodo at https://doi.org/10.5281/zenodo.19914420.

## Supplementary Materials

The following supporting information can be downloaded at: https://www.mdpi.com/article/10.3390/plants15162446/s1, Table S1: Correlation coefficients (Pearson) between all the variables measured, both environmental and physiological (black numbers). Significance levels are shown in red (n = 10). Significant correlations (at least *p* < 0.05) are highlighted in yellow. Variables are defined in the main text.

## Abbreviations

The following abbreviations are used in this manuscript:

AUC_280–400_	Area under the absorbance curve in the interval 280–400 nm
C1	*p*-coumaroylmalic acid
C2	caffeoylmalic acid
C3	feruloylmalic acid
C4	5″-(7″,8″-dihydroxycoumaroyl)-2-caffeoylmalic acid
C5	5″-(7″,8″-dihydroxy-7-O-β-glucosyl-coumaroyl)-2-caffeoylmalic acid
C6	*p*-coumaric acid
C7	ferulic acid
ETR (II) or ETR	Apparent electron transport rate through PSII
F_v_/F_m_	Maximum PSII photochemical quantum yield
Φ_PSII_	Effective PSII photochemical quantum yield
NPQ	Non-photochemical quenching
PAR	Photosynthetically active radiation
PFD	Photon flux density (400–700 nm)
PI	Performance Index
PSII	Photosystem II
SE	Standard error
SUVAC	Methanol-soluble UV-absorbing compounds
UV	Ultraviolet
UV_BE_	Biologically effective UV irradiance
WUVAC	Methanol-insoluble UV-absorbing compounds (mainly cell wall-bound compounds)
