# Supplementary material for: Diel Dynamics of Field Responses to UV and Photosynthetic Radiation in the Aquatic Liverwort Jungermannia eucordifolia and Their Potential Use in UV Biomonitoring
Source: Plants (Basel). 2026 Aug 12;15(16):2446. doi: 10.3390/plants15162446 (PMC13516692; doi:10.3390/plants15162446)
Supplement: Supplementary file 1 [file plants-15-02446-s001.zip › plants-4433612-supplementary.pdf]

Table S1. Correlation coefficients (Pearson) between all the variables measured, both environmental and physiological (black numbers). Significance levels are shown in red (n = 10). Significant correlations (at least p<0.05) are highlighted in yellow. Variables are defined in the main text.

	UVA	PAR	UV_{BE}	UVB_{accum}	UVA_{accum}	PAR_{accum}	UV_{BE_accum}	SUVAC	WUVAC	C1	C2	C3	C4	C5	C6	C7	Temp	NPQ	Φ_{PSII}	F_v/F_m	PI
**UVB**	0.98<br>0.000	0.95<br>0.000	0.99<br>0.000	0.23<br>0.521	0.25<br>0.487	0.28<br>0.441	0.24<br>0.498	0.31<br>0.382	-0.27<br>0.443	-0.16<br>0.660	-0.40<br>0.251	-0.48<br>0.159	-0.16<br>0.663	-0.05<br>0.880	-0.17<br>0.647	-0.16<br>0.656	0.68<br>0.032	0.96<br>0.000	-0.89<br>0.001	-0.83<br>0.003	-0.53<br>0.115
**UVA**		0.99<br>0.000	1.00<br>0.000	0.23<br>0.520	0.25<br>0.481	0.28<br>0.429	0.25<br>0.493	0.46<br>0.184	-0.30<br>0.393	-0.18<br>0.617	-0.40<br>0.259	-0.41<br>0.239	-0.12<br>0.742	-0.09<br>0.813	-0.30<br>0.405	-0.31<br>0.383	0.64<br>0.045	0.98<br>0.000	-0.95<br>0.000	-0.86<br>0.001	-0.66<br>0.038
**PAR**			0.98<br>0.000	0.17<br>0.648	0.19<br>0.605	0.22<br>0.544	0.18<br>0.619	0.52<br>0.119	-0.33<br>0.357	-0.24<br>0.502	-0.42<br>0.223	-0.32<br>0.360	-0.16<br>0.664	-0.17<br>0.630	-0.36<br>0.310	-0.36<br>0.308	0.57<br>0.083	0.98<br>0.000	-0.97<br>0.000	-0.82<br>0.004	-0.69<br>0.028
**UV_{BE}**				0.23<br>0.516	0.25<br>0.478	0.28<br>0.428	0.25<br>0.490	0.41<br>0.238	-0.30<br>0.403	-0.18<br>0.628	-0.40<br>0.253	-0.43<br>0.211	-0.13<br>0.717	-0.08<br>0.835	-0.25<br>0.479	-0.26<br>0.463	0.66<br>0.038	0.98<br>0.000	-0.93<br>0.000	-0.85<br>0.002	-0.62<br>0.056
**UVB_{accum}**					1.00<br>0.000	1.00<br>0.000	1.00<br>0.000	-0.10<br>0.774	-0.32<br>0.372	-0.09<br>0.799	-0.06<br>0.863	-0.49<br>0.150	0.38<br>0.285	0.43<br>0.221	-0.33<br>0.352	-0.46<br>0.178	0.83<br>0.003	0.27<br>0.443	0.00<br>0.991	-0.55<br>0.096	-0.22<br>0.534
**UVA_{accum}**						1.00<br>0.000	1.00<br>0.000	-0.08<br>0.830	-0.32<br>0.362	-0.09<br>0.813	-0.06<br>0.878	-0.49<br>0.155	0.39<br>0.260	0.43<br>0.212	-0.33<br>0.344	-0.47<br>0.172	0.84<br>0.002	0.30<br>0.406	-0.03<br>0.930	-0.56<br>0.089	-0.25<br>0.490
**PAR_{accum}**							1.00<br>0.000	-0.06<br>0.863	-0.32<br>0.368	-0.09<br>0.813	-0.09<br>0.803	-0.47<br>0.171	0.36<br>0.309	0.42<br>0.227	-0.33<br>0.358	-0.49<br>0.147	0.85<br>0.002	0.33<br>0.349	-0.07<br>0.853	-0.60<br>0.069	-0.27<br>0.455
**UV_{BE_accum}**								-0.09<br>0.814	-0.32<br>0.364	-0.09<br>0.809	-0.06<br>0.876	-0.49<br>0.153	0.39<br>0.266	0.43<br>0.214	-0.33<br>0.345	-0.47<br>0.174	0.84<br>0.002	0.29<br>0.418	-0.02<br>0.949	-0.56<br>0.092	-0.24<br>0.503
**SUVAC**									-0.07<br>0.848	-0.05<br>0.897	0.19<br>0.589	0.25<br>0.489	0.31<br>0.386	-0.04<br>0.904	-0.56<br>0.092	-0.35<br>0.324	0.08<br>0.834	0.45<br>0.193	-0.62<br>0.056	-0.25<br>0.483	-0.62<br>0.058
**WUVAC**										0.75<br>0.012	0.63<br>0.049	0.21<br>0.558	0.19<br>0.592	0.59<br>0.072	0.62<br>0.056	0.52<br>0.120	-0.27<br>0.447	-0.38<br>0.273	0.27<br>0.447	0.21<br>0.570	0.46<br>0.179
**C1**											0.67<br>0.033	-0.19<br>0.590	0.45<br>0.193	0.80<br>0.006	0.55<br>0.103	0.43<br>0.217	-0.01<br>0.976	-0.28<br>0.436	0.19<br>0.595	0.01<br>0.987	0.40<br>0.248
**C2**												0.07<br>0.848	0.82<br>0.004	0.74<br>0.015	0.16<br>0.654	0.45<br>0.192	-0.15<br>0.682	-0.49<br>0.150	0.38<br>0.280	0.37<br>0.298	0.33<br>0.352
**C3**													-0.15<br>0.676	-0.31<br>0.376	0.07<br>0.846	-0.04<br>0.913	-0.70<br>0.025	-0.31<br>0.386	0.15<br>0.670	0.59<br>0.072	-0.01<br>0.972
**C4**														0.73<br>0.017	-0.24<br>0.510	0.03<br>0.942	0.24<br>0.497	-0.18<br>0.616	0.16<br>0.659	0.07<br>0.857	0.05<br>0.892
**C5**															0.33<br>0.356	0.28<br>0.431	0.39<br>0.266	-0.16<br>0.668	0.21<br>0.554	-0.17<br>0.639	0.23<br>0.525
**C6**																0.75<br>0.012	-0.26<br>0.470	-0.31<br>0.386	0.32<br>0.375	0.23<br>0.514	0.48<br>0.162
**C7**																	-0.30<br>0.396	-0.38<br>0.275	0.34<br>0.335	0.43<br>0.216	0.51<br>0.136
**Temperature**																		0.65<br>0.042	-0.41<br>0.239	-0.83<br>0.003	-0.31<br>0.386
**NPQ**																			-0.94<br>0.000	-0.84<br>0.003	-0.68<br>0.032
**Φ_{PSII}**																				0.72<br>0.020	0.71<br>0.021
**F_v/F_m**																					0.57<br>0.084
